# Fatal hyperpyrexia caused by xylazine: a case report

**DOI:** 10.3389/fphar.2024.1437960

**Published:** 2024-07-17

**Authors:** Ping Dai, Yanqing Chen, Xianqin Luo, Zhiqiang Zhou, Mengdi Shi, Aerbusili Genjiafu, Xiangdong Jian

**Affiliations:** ^1^ Department of Poisoning and Occupational Diseases, Emergency Medicine, Qilu Hospital, Cheeloo College of Medicine, Shandong University, Jinan, China; ^2^ Emergency Department of Huangdao District Central Hospital, Qingdao, China; ^3^ Emergency Department of Suining Central Hospital, Suining, China; ^4^ Department of Occupational and Environmental Health, School of Public Health, Cheeloo College of Medicine, Shandong University, Jinan, China

**Keywords:** drugs, human, hyperpyrexia, poisoning, xylazine

## Abstract

Xylazine is used in veterinary medicine as a sedative, analgesic, and muscle relaxant. However, in recent decades, it has frequently been detected in illicit drugs. Xylazine poisoning is characterized by depression of the central nervous and cardiovascular systems. Herein, we present a case of a 41-year-old man who not only had severe depression of the central nervous and cardiovascular systems, but also developed hyperpyrexia during the treatment of xylazine poisoning, which led to his death 3 days after poisoning. This case indicates that, in addition to its other effects, xylazine may also cause hyperthermia, which has not yet been reported in humans.

## 1 Introduction

Xylazine, chemical formula C_12_H_16_N_2_S, is a 1,3-thiazide that is structurally similar to phenothiazine (1,4-thiazide) and tricyclic antidepressants. Pharmacologically similar to clonidine, xylazine was originally investigated for the treatment of hypertension; however, it was not clinically approved owing to substantial inhibition of the central nervous and cardiovascular systems (Gupta et al., 2023). Subsequently, it has been used as a veterinary sedative, analgesic, and muscle relaxant (Gupta et al., 2023). However, in 2001, xylazine emerged as a popular illicit drug additive in Puerto Rico (Reyes et al., 2012). In the mid-2010s, it began to spread rapidly across the U.S. drug market, expanding from its initial location in the Northeast to the Rust Belt, South, and West (Zhu, 2023). By 2023, the first death associated with xylazine use was reported in Europe (Rock et al., 2023). Herein, we report a Chinese patient who suffered severe depression of the central nervous and cardiovascular systems, developed hyperthermia during treatment, and subsequently died following xylazine overdose.

## 2 Case description

**FIGURE 1:**
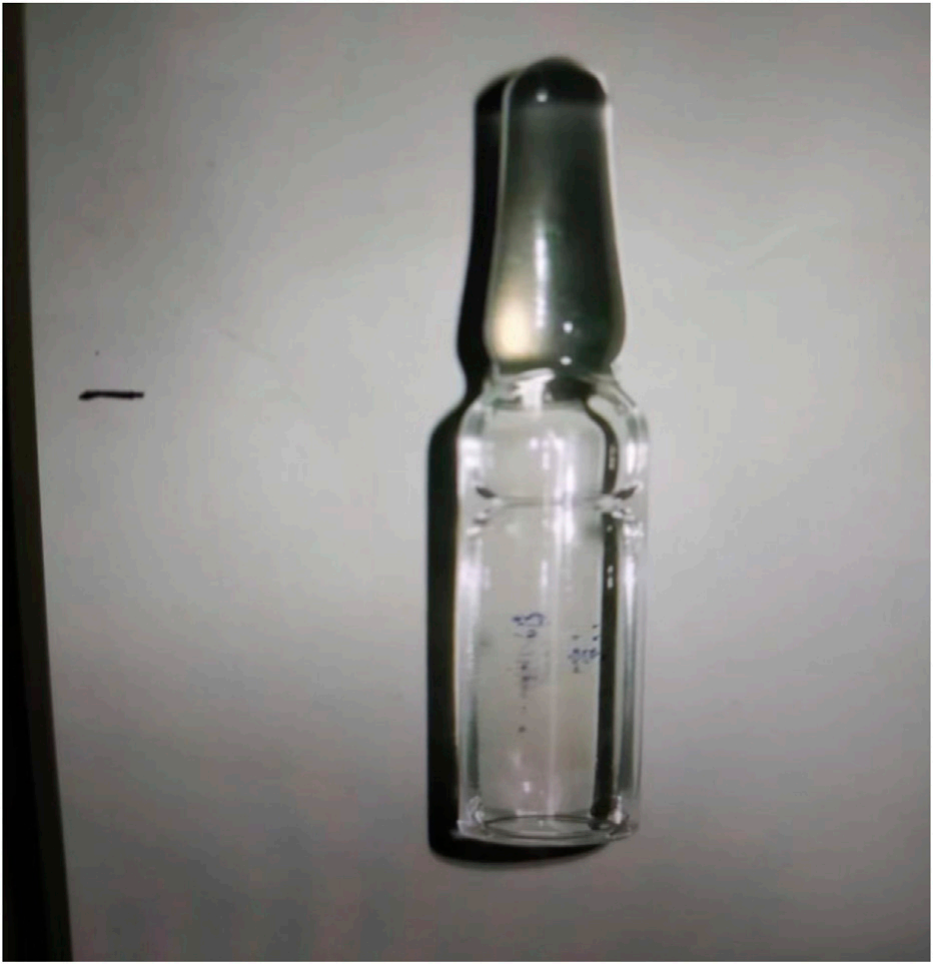
Xylazine ampule discovered beside the patient.

**FIGURE 2:**
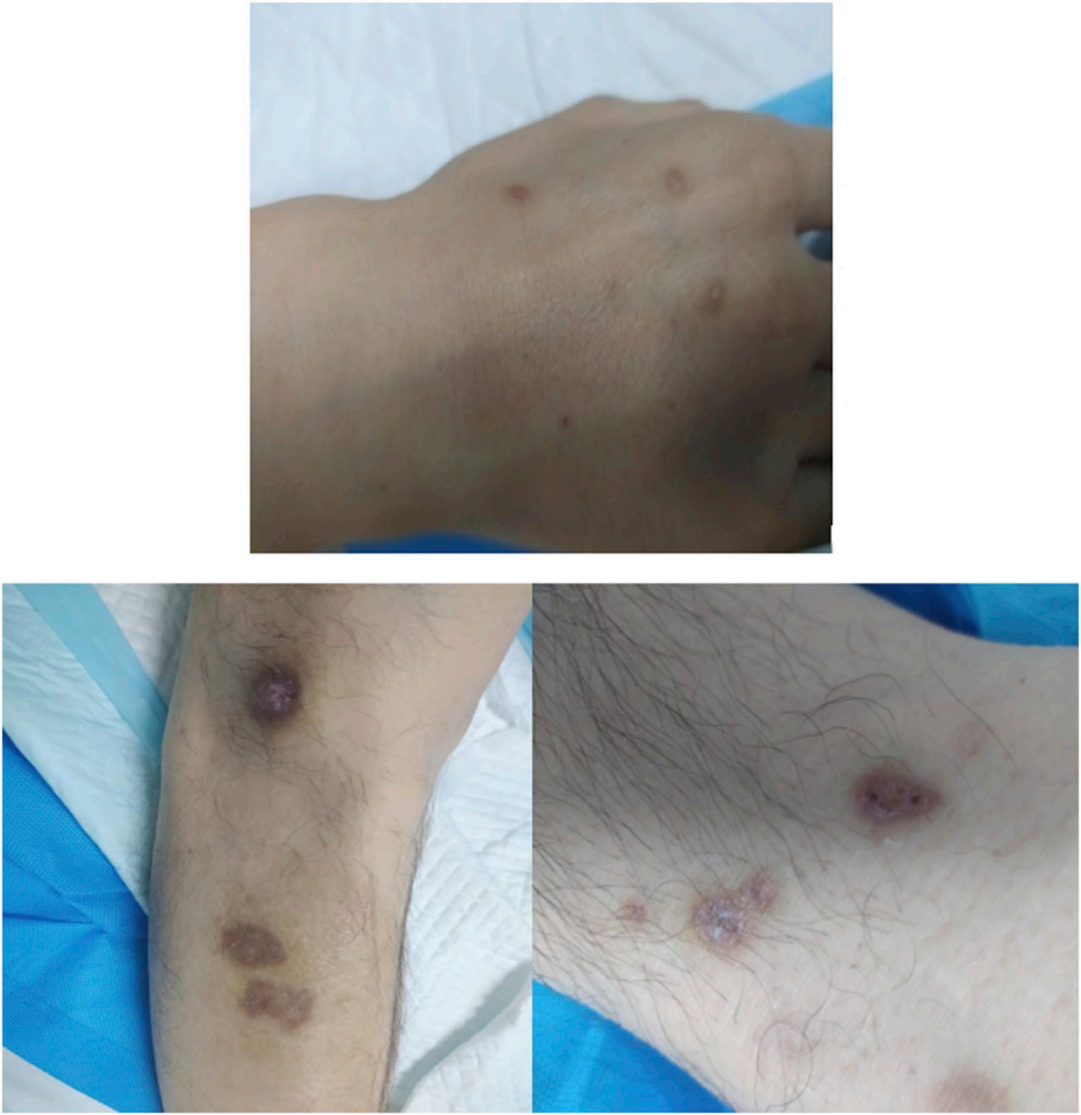
Multiple ulcerated scars on the right arm and the anterior and medial skin of both legs.

A 41-year-old man was transferred to the Poisoning Department of our hospital on 1 June 2022. The patient’s mother reported finding him unconscious at 11 a.m. on that day, with syringes and xylazine ampules beside him (Figure 1). The patient had a history of diabetes mellitus and depression. On admission, body temperature was 36.8°C; heart rate, 78 beats per minute; respiratory rate, 19 breaths per minute; blood pressure, 110/82 mmHg; and Glasgow coma scale score, 2-1–5, 8. The patient had recently lost weight and was uncooperative during the examination. Physical examination revealed multiple ulcerated scars on the right arm and the anterior and medial skin of both legs (Figure 2). The pupils were constricted to 1.5 mm in diameter, equally round, and reactive to light, with sluggish light reflexes. Auscultation of the lungs revealed no moist rales. Initial blood biochemical test results are shown in Table 1. Treatment was initiated immediately and included torsemide (20 mg twice daily), alanyl-glutamine (10 g daily), nalmefene hydrochloride injection (0.1 mg twice daily), insulin, and nutritional support. The family refused blood purification. Subsequently, the patient developed hypotension, and norepinephrine was administered to maintain blood pressure. Approximately 44 h after poisoning, the patient became diaphoretic and febrile up to 39°C. Oxyhemoglobin saturation fell to 68%, and oxygen was administered at 10 L/min by facemask. The heart and respiratory rates increased to 131 beats per minute and 38 breaths per minute, respectively. Blood gas analysis showed a pH of 7.27; PO_2_, 38 mmHg; PCO_2_, 56 mmHg; K+, 3.4 mmol/L; and glucose, 5.1 mmol/L. Therefore, the trachea was intubated and mechanical ventilation was initiated (volume-synchronized intermittent mandatory ventilation mode). Blood cultures were prepared, and flucloxacillin was empirically administered. Rehydration and physical cooling were ineffective. Inflammatory factor levels were as follows: interleukin (IL)-2 receptor, 836 U/mL (reference range, 223–710 U/mL); IL-6, 31.00 pg/mL (reference range, 0–5.9 pg/mL); IL-10, 17.90 pg/mL (reference range, 0–9.1 pg/mL); tumor necrosis factor-α, 8.4 pg/mL (reference value, 0–8.1 pg/mL); and IL-1β and IL-8, within normal limits. Blood gas analysis revealed a pH of 7.33; PO_2_, 89 mmHg; PCO_2_, 33 mmHg; K+, 5.7 mmol/L; lactate, 1.5 mmol/L; and base excess, −7.5 mmol/L. Body temperature remained at approximately 38.6°C after intravenous infusion of 8 mg betamethasone.

**TABLE 1:** Biochemical blood test results.

Biochemical blood indicator	Local hospital		Our department			
	Normal value	06/01 0.5 h after admission	Normal value	06/01 6 h later	06/03 57 h later	06/04 70 h later
WBC (×10^9^/L)	3.5–9.5	8.79	3.5–9.5	10.33	19.94	19.79
NEU (%)	40–75	82.7	40–75	70.60	93.20	92.80
LYM (%)	20–50	12.8	20–50	22.60	3.00	3.80
LYM (#)	1.1–3.2	1.13	1.1–3.2	2.33	0.59	0.75
RBC (×10^12^/L)	4.3–5.8	4.97	4.3–5.8	5.24	4.99	5.83
HGB (g/L)	130–175	144	130–175	153.0	148.0	165.0
PLT (×10^9^/L)	125–350	199	125–350	276	226	263
ALT (U/L)	9–50	154	0–50	155	NA	95
AST (U/L)	NA	NA	17–59	97	NA	78
DBIL (µmol/L)	0.5–6.5	2.07	0–5	0	NA	5.5
IBIL (µmol/L)	1–17	10.35	0–19	12	NA	5.9
CK (U/L)	NA	NA	0.3–4	1.90	1.50	0.7
NT-PROBNP (pg/mL)	NA	NA	<300(acute)	146.30	3812.00	2729.00
BUN (mmol/L)	2.8–7.14	7.1	3.2–7.1	6.1	NA	19.70
Cr (µmol/L)	40–135	69.80	58–133	69	NA	168
PCT (ng/mL)	NA	NA	<0.1	0.044	1.850	NA
GLU (mmol/L)	3.9–6.1	25.31	3.90–6.10	11.63	NA	9.83

ALT, alanine transaminase; AST, aspartate aminotransferase; BUN, blood urea nitrogen; CK, creatine kinase; Cr, creatinine; DBIL, direct bilirubin; GLU, blood glucose; HGB, hemoglobin; IBIL, indirect bilirubin; NEU, neutrophils; PLT, platelets; RBC, red blood cells; WBC, white blood cells; NT-PROBNP, N-terminal pro-brain natriuretic peptide; PCT, procalcitonin; LYM, lymphocyte; NA, missing value.

On the third day, the patient’s body temperature was 41°C; heart rate, 140 beats per minute; and blood pressure, 90/70 mmHg. He remained febrile despite application of a cooling blanket, and empiric moxifloxacin and flucloxacillin were administered. The patient’s temperature peaked at 41.2°C, and he died of hyperpyrexia. The blood culture later showed no aerobic or anaerobic bacteria.

## 3 Discussion

Xylazine, an α-2 adrenoceptor agonist, reduces the release of norepinephrine and dopamine from the central nervous system, resulting in sedative, neuromuscular, and analgesic effects. The mechanism of action may also involve H2-histaminergic, serotonergic, dopaminergic, and opioid receptors (Kitzman et al., 1982; Capraro et al., 2001; Hoffmann et al., 2001; Moore et al., 2003). The route of xylazine poisoning is usually intravenous, intramuscular, or subcutaneous injection; however, oral, ocular, and inhalational exposures occur. In the case of overdose, the patient presents with hyperglycemia and central nervous system, respiratory, and cardiovascular depression symptoms such as vertigo, disturbance of consciousness, and hypotension (Capraro et al., 2001; Hoffmann et al., 2001; Elejalde et al., 2003; Velez et al., 2006; Ball et al., 2022). Long-term use can cause skin ulcers, drug dependence, and physical decline (Ruiz-Colón et al., 2014; Mulders et al., 2016). Xylazine is metabolized by hepatic cytochrome P450 and primarily eliminated by the kidneys (Hoffmann et al., 2001). The effects of xylazine in animals generally last 4 h; however, in reported human overdoses, the duration of effects can be 8–72 h (Velez et al., 2006).

Our patient presented with disturbance of consciousness and hypotension, with sweating and temperatures as high as 39°C occurring approximately 44 h after poisoning. Owing to fever, we first considered infection; the patient’s procalcitonin level was normal on admission but increased with an increase in body temperature, and sepsis and a moderate systemic inflammatory response were suspected (Wanqing et al., 2019). At the time of fever, the patient’s IL-6 level was 31.00 pg/mL, indicating the presence of mild inflammation or infection (Feng et al., 2016). However, anti-infection, rehydration, and antipyretic treatments were ineffective; despite administering betamethasone (8 mg), the body temperature remained high. Subsequently, we intensified the anti-infective therapy with moxifloxacin, but the temperature continued to rise. The patient died 1 day later after reaching a temperature of 41.2°C.

Inflammation was not severe at the time of fever, and difficulties in controlling the body temperature with antibiotics and glucocorticoids forced us to consider alternative causes. Malignant hyperthermia (MH) is a clinical syndrome with autosomal dominant inheritance as the main mode of inheritance. Its typical clinical manifestations mostly occur after the application of volatile inhalation anesthetics, such as halflurane, isoflurane, and/or succinylcholine (Kaur et al., 2019). Clinically, patients with paroxysmal MH are characterized by a sharp increase in core body temperature and severe acidosis, which may further develop into rhabdomyolysis (Kaur et al., 2019). However, in our case, creatine kinase levels were within the normal range, and there was no generalized muscle rigidity, indicating that xylazine-induced MH was unlikely. Because the body temperature of certain animals increases after xylazine administration (Hopkins, 1972), we believe that xylazine toxicity cannot be ruled out as a cause of the high fever in this case. How xylazine causes hyperthermia is unknown; however, its pharmacological analog, clonidine, impairs thermoregulation in guinea pigs (*Cavia porcellus*) by stimulating preoptic area prostaglandin E2 release (Feleder et al., 2004). This release promotes central thermogenesis, increasing body heat production. Additionally, α-2 adrenergic agonists also promote peripheral vasoconstriction (Maze and Tranquilli, 1991) and prevent excessive body surface heat loss (Flavahan, 1991). Regarding the delayed presentation of hyperpyrexia, Feleder et al. (Feleder et al., 2004) reported that Clonidine induced a gradual, significant decrease in the core temperature (Tc) in guinea pigs, followed by a rapid, significant increase. Unfortunately, computed tomography, genetic testing, and other ancillary studies were not performed after fever onset because the patient’s family did not provide consent.

With no available antidote for xylazine poisoning, symptomatic and supportive treatment remains a priority, with maintenance of respiratory function and blood pressure as the main goals. Although α-adrenergic antagonists such as phentolamine, yohimbine, and metazoline have been proposed as antidotes, they have not been tested in humans (Capraro et al., 2001; Elejalde et al., 2003). The substantial predicted volume of distribution implies that hemodialysis would not effectively enhance elimination. As the stomach is also involved in the metabolism of xylazine (Hoffmann et al., 2001), gastric lavage may represent an important detoxification measure.

## Data Availability

The original contributions presented in the study are included in the article/Supplementary material, further inquiries can be directed to the corresponding author.

## References

[B1] Ball N. S. Knable B. M. Relich T. A. Smathers A. N. Gionfriddo M. R. Nemecek B. D. (2022). Xylazine poisoning: a systematic review. Clin. Toxicol. (Phila). 60, 892–901. 10.1080/15563650.2022.2063135 35442125

[B2] Capraro A. J. Wiley J. F. Tucker J. R. (2001). Severe intoxication from xylazine inhalation. Pediatr. Emerg. Care 17, 447–448. 10.1097/00006565-200112000-00012 11753193

[B3] Elejalde J. I. Louis C. J. Elcuaz R. Pinillos M. A. (2003). Drug abuse with inhalated xylazine. Eur. J. Emerg. Med. 10, 252–253. 10.1097/00063110-200309000-00022 12972909

[B4] Feleder C. Perlik V. Blatteis C. M. (2004). Preoptic alpha 1- and alpha 2-noradrenergic agonists induce, respectively, PGE2-independent and PGE2-dependent hyperthermic responses in Guinea pigs. Am. J. Physiol. Regul. Integr. Comp. Physiol. 286, R1156–R1166. 10.1152/ajpregu.00486.2003 14962823

[B5] Feng M. Sun T. Zhao Y. Zhang H. (2016). Detection of serum interleukin-6/10/18 levels in sepsis and its clinical significance. J. Clin. Lab. Anal. 30, 1037–1043. 10.1002/jcla.21977 27184083 PMC6807223

[B6] Flavahan N. A. (1991). The role of vascular alpha-2-adrenoceptors as cutaneous thermosensors. Physiology 6, 251–255. 10.1152/physiologyonline.1991.6.6.251

[B7] Gupta R. Holtgrave D. R. Ashburn M. A. (2023). Xylazine - medical and public health imperatives. N. Engl. J. Med. 388, 2209–2212. 10.1056/NEJMp2303120 37099338

[B8] Hoffmann U. Meister C. M. Golle K. Zschiesche M. (2001). Severe intoxication with the veterinary tranquilizer xylazine in humans. J. Anal. Toxicol. 25, 245–249. 10.1093/jat/25.4.245 11386637

[B9] Hopkins T. J. (1972). The clinical pharmacology of xylazine in cattle. Aust. Vet. J. 48, 109–112. 10.1111/j.1751-0813.1972.tb02228.x 5024310

[B10] Kaur H. Katyal N. Yelam A. Kumar K. Srivastava H. Govindarajan R. (2019). Malignant hyperthermia. Mo Med. 116, 154–159. 31040503 PMC6461318

[B11] Kitzman J. V. Booth N. H. Hatch R. C. Wallner B. (1982). Antagonism of xylazine sedation by 4-aminopyridine and yohimbine in cattle. Am. J. Vet. Res. 43, 2165–2169. 6131631

[B12] Maze M. Tranquilli W. (1991). Alpha-2 adrenoceptor agonists: defining the role in clinical anesthesia. Anesthesiology 74, 581–605. 10.1097/00000542-199103000-00029 1672060

[B13] Moore K. A. Ripple M. G. Sakinedzad S. Levine B. Fowler D. R. (2003). Tissue distribution of xylazine in a suicide by hanging. J. Anal. Toxicol. 27, 110–112. 10.1093/jat/27.2.110 12670006

[B14] Mulders P. van Duijnhoven V. Schellekens A. (2016). Xylazine dependence and detoxification: a case report. Psychosomatics 57, 529–533. 10.1016/j.psym.2016.05.001 27480943

[B15] Reyes J. C. Negrón J. L. Colón H. M. Padilla A. M. Millán M. Y. Matos T. D. (2012). The emerging of xylazine as a new drug of abuse and its health consequences among drug users in Puerto Rico. J. Urban Health 89, 519–526. 10.1007/s11524-011-9662-6 22391983 PMC3368046

[B16] Rock K. L. Lawson A. J. Duffy J. Mellor A. Treble R. Copeland C. S. (2023). The first drug-related death associated with xylazine use in the UK and Europe. J. Forensic Leg. Med. 97, 102542. 10.1016/j.jflm.2023.102542 37236142

[B17] Ruiz-Colón K. Chavez-Arias C. Díaz-Alcalá J. E. Martínez M. A. (2014). Xylazine intoxication in humans and its importance as an emerging adulterant in abused drugs: a comprehensive review of the literature. Forensic Sci. Int. 240, 1–8. 10.1016/j.forsciint.2014.03.015 24769343

[B18] Velez L. I. Shepherd G. Mills L. D. Rivera W. (2006). Systemic toxicity after an ocular exposure to xylazine hydrochloride. J. Emerg. Med. 30, 407–410. 10.1016/j.jemermed.2006.02.042 16740450

[B19] Wanqing M. Yannan Z. Yanyan H. (2019). The clinical research progress of procalcitonin(PCT) in the diagnosis and treatment of sepsis. Fudan J. Med. Ed. 46, 103–107. 10.3969/j.issn.1672-8467.2019.01.018

[B20] Zhu D. T. (2023). Public health impact and harm reduction implications of xylazine-involved overdoses: a narrative review. Harm Reduct. J. 20, 131. 10.1186/s12954-023-00867-x 37700329 PMC10498612

